# Gross motor performance of infants with an at‐home wearable measurement and the Alberta Infant Motor Scale: A concurrent validity study

**DOI:** 10.1111/dmcn.70175

**Published:** 2026-02-16

**Authors:** Manu Airaksinen, Taru Palsa, Arto Hautala, Marike Boonzaaijer, Leena M. Haataja, Sampsa Vanhatalo

**Affiliations:** ^1^ BABA Center, Pediatric Research Center, New Children's Hospital Helsinki University Hospital Helsinki Finland; ^2^ Department of Physiology University of Helsinki Helsinki Finland; ^3^ Department of Child and Adolescent Psychiatry, University Hospital Heidelberg, Heidelberg University German Center for Mental Health (DZPG) Heidelberg Germany; ^4^ Department of Pediatric Neurology, Children's Hospital Helsinki University Hospital and University of Helsinki Helsinki Finland; ^5^ Faculty of Sport and Health Sciences University of Jyväskylä Jyväskylä Finland; ^6^ Growing, Moving and Thriving Together Research Group, Centre of Expertise Healthy and Sustainable Living HU University of Applied Science Utrecht the Netherlands

## Abstract

**Aim:**

To evaluate the concurrent validity of assessing infants' gross motor performance with an at‐home wearable measurement versus the Alberta Infant Motor Scale (AIMS).

**Method:**

This observational study used a new multi‐sensor wearable Motor Assessment of Infants with a JUmpsuit (MAIJU) for 67 at‐home measurements of 42 infants (60% males; mean age 11.5 months, SD 3.7 months, range 4–18 months). The study collates a normative cohort of typically developing volunteers (*n* = 17, 65% males; 27 measurements, mean age 11.8 months, SD 3.1 months, range 7–18 months) and a clinical cohort recruited from a neurodevelopmental follow‐up clinic (*n* = 25, 56% males; mean age 11.2 months, SD 3.8 months, range 4–17 months). We correlated the expert‐assessed total AIMS score to a holistic assessment of motor development (the BABA Infant Motor Score [BIMS]) from the MAIJU measurements. Additionally, detailed MAIJU‐derived metrics were compared to the AIMS scores, to train machine learning‐based AIMS predictions.

**Results:**

There was a very strong correlation (Spearman's rank correlation coefficient [rho] = 0.97, *p* < 10^−40^) between BIMS and AIMS. Centile‐based detection of low‐performing individuals (cut‐offs of 5% and 10%) showed high agreement between AIMS and BIMS (Cohen's kappa = 0.81). Performance of a machine learning‐based AIMS prediction from the wearable data was comparable (rho = 0.96, *p* < 10^−37^) to direct use of the BIMS score.

**Interpretation:**

Assessing motor performance with scalable, at‐home wearable measurements is highly comparable to the widely used AIMS assessment. An objective, quantitative, and expert‐independent motor assessment holds promise for providing benchmarks in geographically distributed health care or clinical trials.

## Graphical abstract

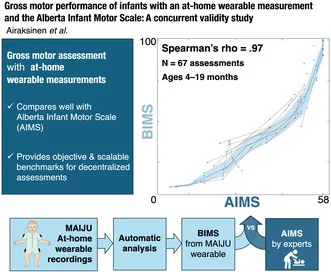

**This original article is commented by Murray on pages** 1337–1338 **of this issue.**

**Abbreviations**
AIMS: Alberta Infant Motor Scale
BIMS: BABA Infant Motor Score
GMP: gross motor performance
MAIJU: Motor Assessment of Infants with a JUmpsuit

**What this paper adds**

- Assessment of infants' motor performance can be done at home using multi‐sensor wearable measurements.
- Motor assessments using a multi‐sensor wearable system (MAIJU) or the Alberta Infant Motor Scale (AIMS) are highly comparable.
- Developmental trajectories of posture and movement are related to the AIMS scores.
- Wearable‐based metrics can track motor development beyond the upper limit of the AIMS.

Assessing an infant's gross motor performance (GMP) is a key aspect of developmental follow‐up in both typically developing infants^1^ and the evaluation of infants with neurological compromise.^2^ An infant's GMP provides efficient surrogate measures of wider neurodevelopment, with significant links to earlier adversities, environmental and other acquired effects, and later neurodevelopmental compromise.^3^, ^4^ Because of variability in motor development between and within infants, it is often essential to assess GMP repeatedly in a longitudinal manner over the course of several months.^4^ These assessments typically rely on parental interviews about achieving motor milestones; brief complementary observations may be feasible in paediatric clinics.^5^ Trained clinical professionals can also use standardized and validated neurodevelopmental assessment scales based on fixed sets of items to be observed or tested during the appointment. While parental interviews are by default subjective and potentially biased, clinical assessment scales can be more harmonized and standardized. However, clinical assessments are typically used during clinical visits, which cannot fully capture an infant's true neurobehavioural performance at home, this being the ecologically most relevant environment from the child's perspective.

The biggest challenges, or ambiguities, in assessing early motor skills are (1) a very large intraindividual and interindividual variability^6^, ^7^ caused by both the child and environmental factors;^4^ (2) logistic problems in assessing infants in their own environment for an extended period of time to improve ecological validity; (3) current methods that focus on observing motor skills as achieved milestones, or maximal performance, thus ignoring the typical behavioural preference in using a wide repertoire of movements that characterizes the developing motor performance;^8^ (4) neurodevelopment that should be assessed longitudinally rather than using point measures, both to mitigate the aforementioned ambiguities and to capture the individual's developmental trajectory.

Improving neurodevelopmental assessment calls for methods that are genuinely objective, quantifiable, and scalable for longitudinal follow‐up. Intuitively, assessing an infant's spontaneous daily activity at home would provide the optimal view of their GMP.^9^, ^10^ Recent development of a multi‐sensor wearable suit, the Motor Assessment of Infants with a JUmpsuit (MAIJU), and its fully automated machine learning‐based analyses,^8^, ^11^, ^12^, ^13^ has made it possible to quantify an infant's GMP at home using metrics that are fully objective, accurate, and can be explained intuitively;^8^, ^11^ however, their wider uptake in the professional community calls for validation by comparing them with standardized assessment scales that are in use worldwide.

In this study, we set out to assess the concurrent validity of the at‐home MAIJU measurements compared with the widely used Alberta Infant Motor Scale (AIMS).^14^ For added transparency and understanding, we also systematically compared the detailed MAIJU‐derived metrics with the AIMS scores. Because there is no ‘criterion standard’ for GMP, we chose AIMS as the benchmark as it is a straightforward method with high interrater reliability,^15^, ^16^, ^17^ which is used internationally for longitudinal follow‐up with established normative charts for different cultures.^5^, ^18^, ^19^ AIMS measures three components of GMP, that is, weight‐bearing, posture, and antigravity movement using 58 items, observing infants' spontaneous movements while prone, supine, sitting, and standing. The postural context subdivisions in AIMS can also support a transparent item‐level comparison between AIMS scores and the metrics from the measurements obtained with the wearable suit.

## METHOD

**Figure 1:**
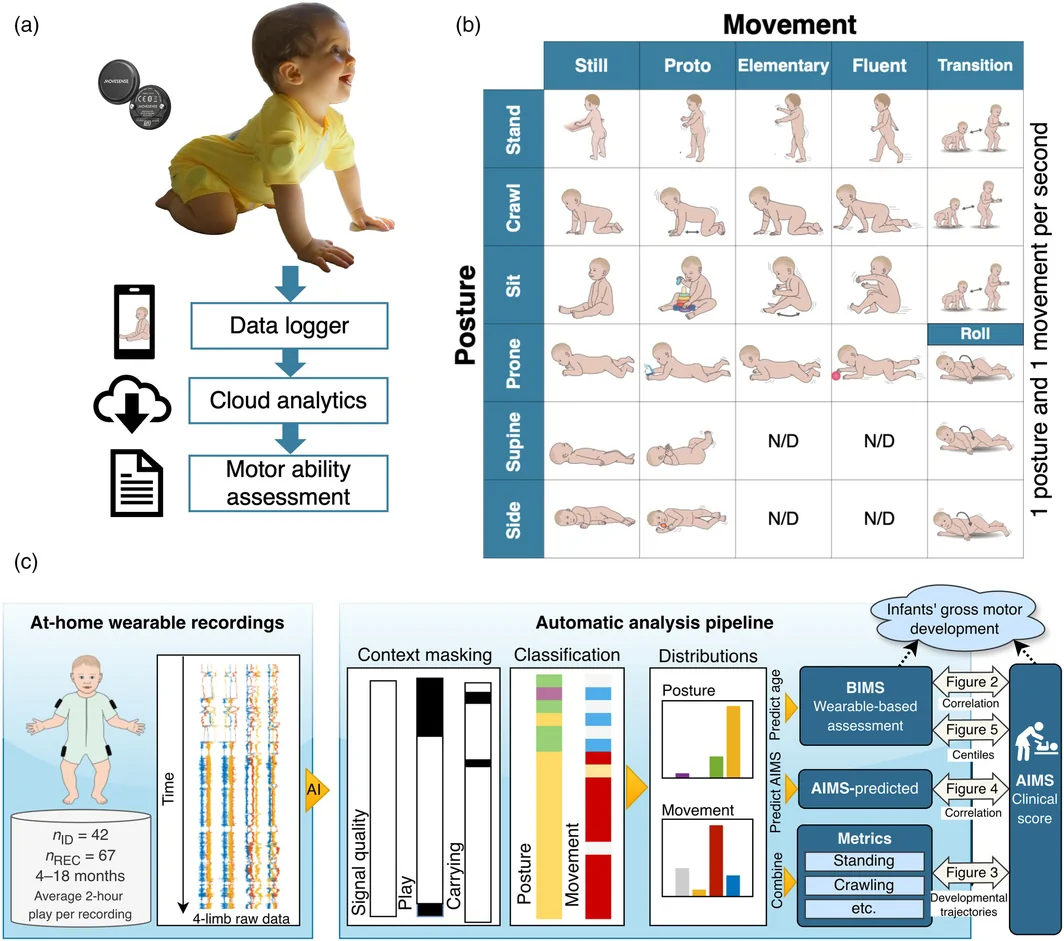
Overview of the study design. (a) The infants were measured at home using the MAIJU wearable combined with a fully automated analysis pipeline. (b) The pipeline provides second‐by‐second detection of key postures (rows) and movements (columns) during the whole measurement. (c) The total occurrence of each posture and the holistic BIMS index (MAIJU metric) are used for comparison with the AIMS (total scores, development, and centiles). Abbreviations: AI, artificial intelligence; AIMS, Alberta Infant Motor Scale; BIMS, BABA Infant Motor Score; MAIJU, Motor Assessment of Infants with a JUmpsuit; N/D not determined.

We used our recently developed and validated multi‐sensor wearable MAIJU (Figure 1)^8^, ^11^, ^12^, ^20^ and its fully automated analysis pipeline to obtain high‐resolution quantification of an infant's posture and movement (MAIJU metrics). These metrics were compared to the AIMS assessment performed by a trained paediatric physiotherapist. The study was reviewed by the Ethics Committee and approved by the New Children's Hospital in Helsinki University Hospital, Helsinki, Finland (no. HUS/80/2021); written informed consent was obtained from the legal guardians.

### Participants and AIMS assessment

We used MAIJU measurements from 67 sessions in 42 infants (60% males; mean age 11.4 months, SD 3.6 months, range 4–18 months; Figure S1), who were originally recruited for a larger longitudinal study (PILKE study),^8^, ^12^ to develop a wearable methodology for studying GMP and its relationship to cognitive performance^21^ or sleep at home.^22^ The present data set includes an ad hoc convenience sample of the larger PILKE study population because the AIMS assessment was not originally included in the PILKE protocol. The sample contains infants from both our normative cohort and the cohort with diagnosed or suspected developmental delay (the clinical cohort). The normative cohort consisted of 17 typically developing volunteers (65% males; 27 measurements, mean age 11.8 months, SD 3.1 months, range 7–18 months). We used the following inclusion criteria for the normative cohort: (1) the infant was born at full term and no neurological or other significant medical issues since pregnancy were noted; (2) parents showed sufficient language competence to consent in Finnish; (3) no developmental concerns during the study period were identified, as documented by inspecting the reports of postnatal follow‐up visits systematically done for all children in Finland according to the nationwide health care guidelines. The clinical cohort (*n* = 25, 45 measurements, 56% males; mean age 11.2 months, SD 3.8 months, range 4–17 months) was recruited from the neurodevelopmental follow‐up clinic of the University Hospital of Helsinki. Infants had been referred for neurological evaluation because of an identified or suspected developmental risk (e.g. preterm birth, birth asphyxia). Eight of 25 infants received a neurological diagnosis: three infants had spastic unilateral cerebral palsy; two infants had a significant language delay; one infant had severe motor and cognitive delay; one infant had autism spectrum disorder; and one infant had an unspecified syndrome by 2 years of age. Seventeen of 25 infants had transient motor or tone asymmetries that normalized at the latest by 2 years of age.

The AIMS assessments were done by an experienced paediatric physiotherapist (TP) with more than 12 years' experience of early neurodevelopmental assessments. The assessments were done either as real‐time observation or by assessing AIMS from videos recorded by the parents according to a structured protocol previously validated to provide AIMS as reliably as a direct visual observation.^16^ We used the AIMS total score centiles based on the published Canadian reference population.^23^ To maximize the situational independence of the AIMS and MAIJU assessments, AIMS was not assessed at the same time as the wearable measurements, but it was assessed within 2 weeks of the MAIJU measurement. Our post hoc analyses showed that the time interval between AIMS and MAIJU assessments did not introduce any systematic bias when they were compared (see Figure S2).

### Wearable measurements

The wearable suit (Figure 1a) is a garment with four movement sensors attached to standardized limb locations.^11^ The present data set included accelerometer and gyroscope data collected at 52 Hz (1024 samples per second from the 4‐sensor set‐up), and transmitted over Bluetooth connection to a nearby mobile device. The suit was delivered from the hospital to the home setting using local courier services. Parents were advised to put the suit on the infant and encourage the infant to be spontaneously active (‘free play’) for at least an hour, and preferably for the rest of the day.^20^ In the present cohort, we did not observe the child's performance during measurements taken while wearing the suit as being substantially different from their typical behaviour. An infant's mood may affect behaviour, but the at‐home measurement sessions were long enough to allow capturing moments with the infant in a better mood, that is, the child's typical performance during free play. The measurement sessions were rescheduled by the parents if the child was acutely ill. The median duration of free play in the MAIJU measurements was 65 minutes (mean = 91.1 minutes; interquartile range = 54–119 minutes; range = 15–326 minutes).

### Analysis of the wearable data

On returning to the laboratory, data from the wearable suit were uploaded using a mobile device to a fully automated and previously validated analysis pipeline on the ‘Babacloud’ computational server.^11^, ^20^ Briefly, the analysis pipeline detected both the infant's posture (seven categories: supine, prone, side left, side right, crawl posture, sitting, standing) and movement (nine categories: still, roll left, roll right, pivot left, pivot right, proto, elementary, fluent, transition) at a second‐level time resolution. These posture and movement time series were also used for automated identification of segments with free play and carrying. In addition, we computed the holistic estimate of motor development, that is, the BABA Infant Motor Score (BIMS), which was developed by combining an infant's posture and movements during a wearable measurement session.^11^ Technically, it predicts the most likely age (based on normative data) to generate the given observation; thus, it is also called developmental age prediction.^12^ The BIMS index is normalized to 0 to 100 as follows: 0 denotes motor performance with supine‐dominant preference without rolling; 100 denotes motor performance with fluent walking as the dominant mode of locomotion. In typically developing infants, the BIMS correlates strongly with chronological age.^8^, ^12^ The BIMS centiles (BIMS% was obtained from a previously published independent data set of *n* = 463 MAIJU measurements: age range = 4.5–19.0 months) from 97 typically developing infants were obstained.^8^

### Statistical methods and comparisons between MAIJU metrics and AIMS scores

**Figure 2:**
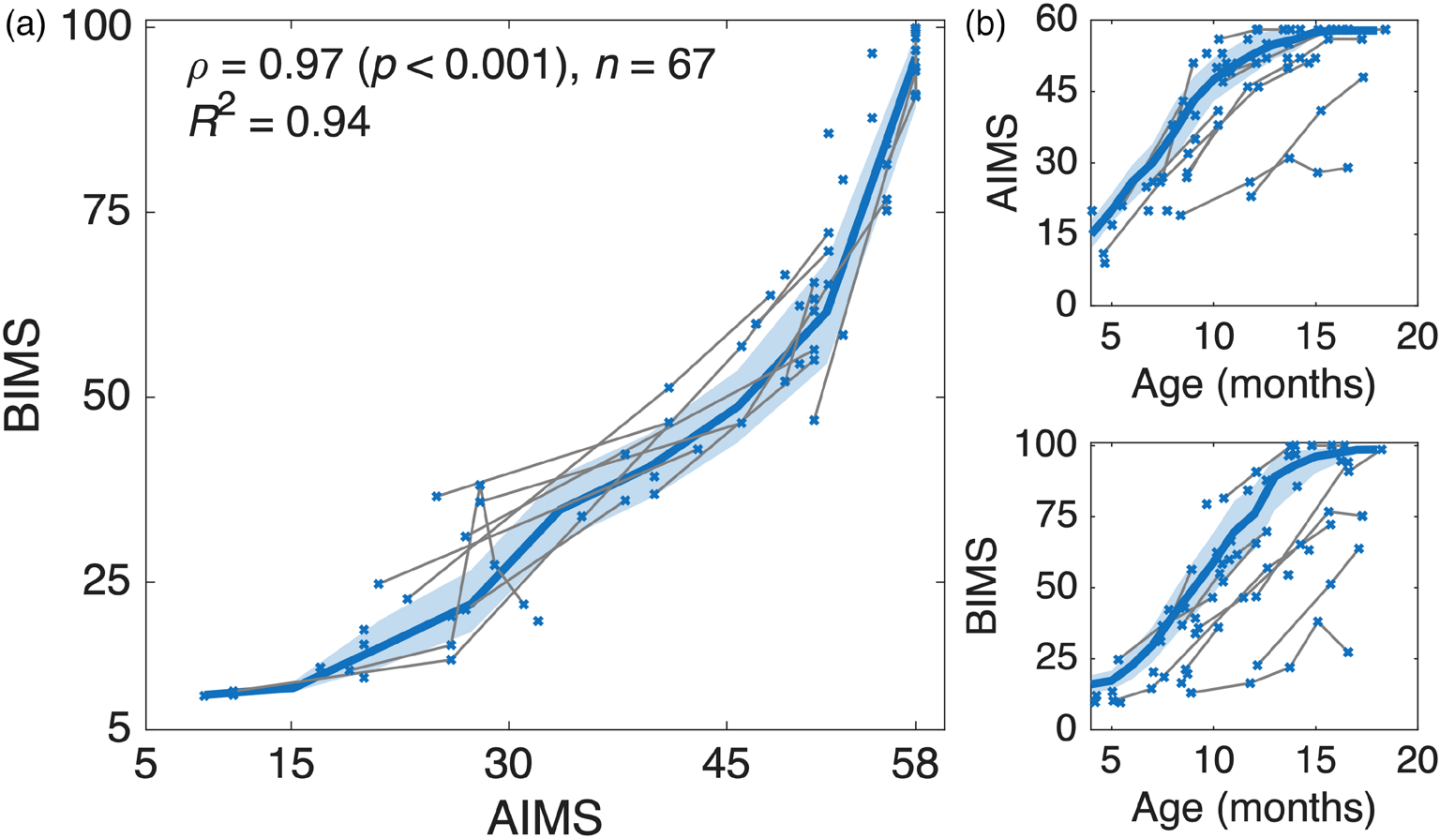
Direct comparison between AIMS and BIMS. (a) The scatter plot shows the total AIMS (*x* axis, range 0–58) and BIMS (*y* axis, range 0–100) scores. The blue line denotes the group median trajectory and its confidence interval (interquartile range, light blue). *R* ^2^ is the coefficient of determination between the median line and the data points. Note how BIMS increases after AIMS has reached its maximum. (b) BIMS and AIMS results plotted against age at assessment (blue crosses). Note how the AIMS scores reach saturation several months before the BIMS scores. For visual reference, the dark and light blue shading denotes the normative centile trajectories for the 25th, 50th, and 75th centiles; the lines between the crosses denote infants who were assessed repeatedly. Abbreviations: AIMS, Alberta Infant Motor Scale; BIMS, BABA Infant Motor Score.

The automatically generated wearable results, collectively called MAIJU metrics, were compared to the AIMS total scores during several phases: (1) we correlated the BIMS results of each measurement directly with the total AIMS score using Spearman's rho (Figure 2). Comparison of BIMS with the total AIMS score was reasoned using the prior literature, which shows that BIMS is a holistic measure that strongly correlates with an infant's progress in motor performance and milestones;^8^ (2) we plotted the individual MAIJU metrics (occurrence of key posture + movement combinations) as a function of AIMS. This analysis was heuristically reasoned using the AIMS rationale to score GMP according to posture; we hypothesized that individual MAIJU metrics should exhibit ‘motor growth charts’ linked tightly to the AIMS scores; (3) we tested predicting an infant's AIMS score from the MAIJU measurements using a Gaussian process regression model and the same gross motor features as in the BIMS;^11^ we studied this with information from both posture and movement, and with information about posture alone. Prediction performance was assessed with leave‐one‐subject‐out cross‐validation (LOSO; at the individual level, that is, all of each individual's measurements are held out on a single testing fold); (4) we compared the population centiles between AIMS^23^ and BIMS, motivated by common clinical practice of using low centiles (e.g. below 5%) as a cut‐off for prompting therapeutic interventions in neurodevelopmental care. Agreement rates between the AIMS and BIMS centile cut‐off groups were assessed for the 5% and 10% groups using accuracy and Cohen's kappa metrics. We report accuracy and Cohen's kappa for all measurements together. Additionally, we report weighted measures of accuracy and kappa, where we controlled repeated measurements from the same individual by scaling each measurement's contribution to the overall score by 1/*n*, where *n* is the number of repeated measurements for a given individual.

**Figure 3:**
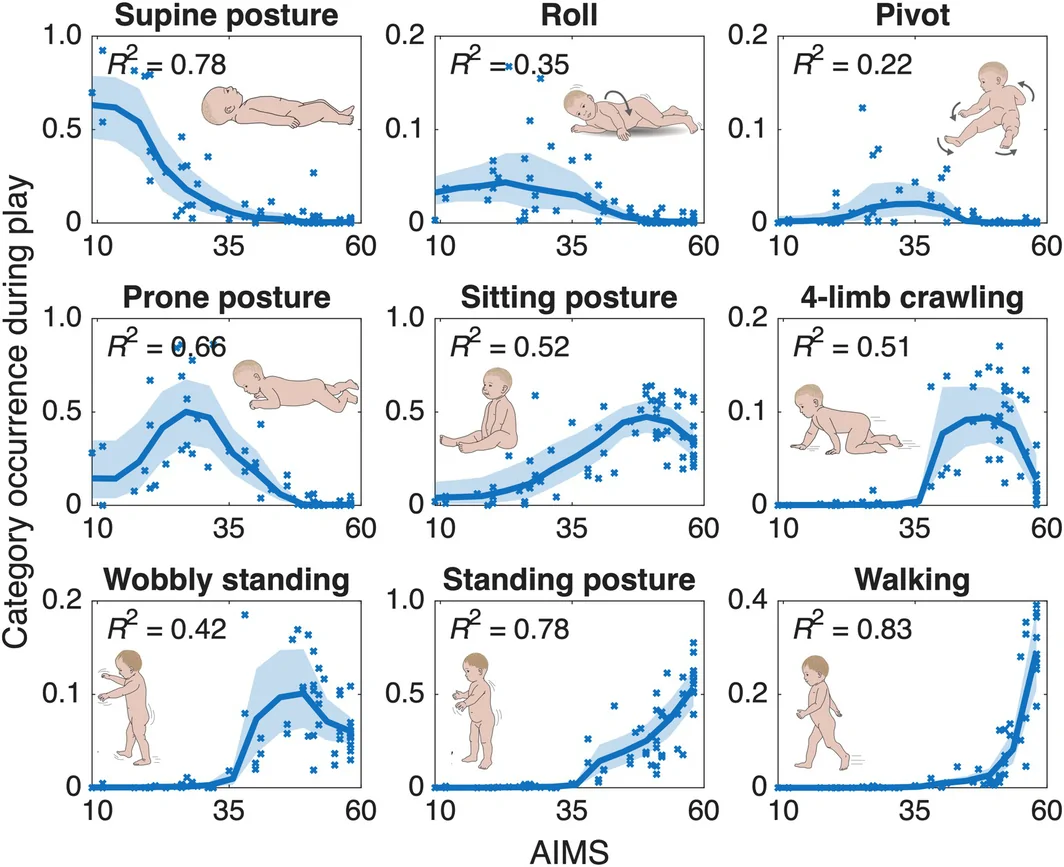
Developmental trajectories of posture + movement combinations as a function of total AIMS score (*x*‐axis). Note the clear trajectories with intuitively expected declining, transient, and increasing shapes. The blue crosses denote individual measurements; dark blue denotes the population median, while the blue shading denotes the interquartile range. *R* ^2^ is the coefficient of determination for the median trajectory in modelling the data points (i.e. the proportion of explained variance in MAIJU metrics as a function of AIMS). Abbreviations: AIMS, Alberta Infant Motor Scale; MAIJU, Motor Assessment of Infants with a JUmpsuit.

The coefficient of determination (*R* ^2^) in Figure 3 was used to measure the proportion of variance in the raw data points (individual points) that is explained by the BIMS‐dependent and AIMS‐dependent median trajectories (the thick blue line; obtained with Beta distribution modeling, see Figure S3).

All statistical analyses were done using custom‐made scripts in MATLAB v2025a (MathWorks, Natick, MA, USA).

### Additional analyses

Several ad hoc analyses were performed to exclude potential confounders: (1) AIMS and BIMS growth charts were constructed to confirm the overall idea that they exhibited clear age‐dependent trajectories; (2) akin to the main analysis, we also plotted BIMS values against the occurrence of key MAIJU metrics (posture and movement) to confirm that both AIMS and BIMS follow comparable functional trajectories; (3) we assessed the correlation between an infant's age at measurement and the AIMS% or BIMS% respectively, to confirm that the slightly different age at BIMS and AIMS measurements does not bias their comparison (Figure S2). Finally, we also compared AIMS versus BIMS correlations in groups of in‐person versus video‐based AIMS; we found no difference (Figure S4).

## RESULTS

All 67 MAIJU measurements were technically successful, from data collection to fully automated processing via the analysis pipeline, providing metrics for comparison with the AIMS scores. Likewise, all videos were of good enough technical quality; none were excluded because of violation of procedures. Therefore, no data were excluded from further analyses.

### Comparison between BIMS and AIMS

We first assessed how well the wearable‐derived holistic index of motor development, that is, the BIMS, correlated with the total AIMS score. Correlation across the full AIMS range (rho = 0.97, *p* < 10^−40^; *n* = 67; Figure 2a) was very high. In addition, the BIMS in some assessments increased even when the AIMS total score had reached its maximum value of 58. Plotting BIMS and AIMS against the infant's current age yielded clear growth charts with comparable age correlation for both measures (*r* = 0.74 in both; Figure 2b). Individual cases with significant motor delay are clearly seen in both charts. Notably, AIMS values reached their ceiling at a younger age compared to BIMS, directly supporting the observation in Figure 2a. There was no significant correlation between the duration of measured free play and the infant's age (rho = −0.12, *p* = 0.33) or the difference in AIMS versus BIMS centiles (AIMS%–BIMS%; rho = 0.11, *p* = 0.38).

### Comparison of AIMS with MAIJU‐derived motor metrics

The underlying rationale in AIMS splits motor performance according to posture; AIMS recognizes developing weight‐bearing and antigravity movements. In the MAIJU video recordings, the analysis pipeline followed a comparable yet independent rationale with distinct posture and incremental movement qualities within each posture (Figure 1b). Thus, we reasoned that occurrence of specific posture + movement combinations should have clear developmental trajectories as a function of the total AIMS scores. As shown in Figure 3, the nine posture + movement combinations showed three basic types of trajectories: (1) a declining trajectory was seen with supine posture and rolling; (2) an intermittent rise and fall (‘hump’) was seen with prone lying, pivoting, four‐limb crawling, sitting, and wobbly standing, each at a different range of AIMS scores; and (3) a rising trajectory was clear in standing posture and walking, starting to rise at approximately 35 for AIMS (total score) and approximately 50 for AIMS respectively. The AIMS total scores correlated strongly with the expected functional performance level. For instance, infants with AIMS greater than 30 are expected to start crawling, spend playtime while sitting, and gradually start to get up to a standing posture. Meanwhile, infants with an AIMS greater than 40 are expected to spend increasing time in standing posture, while an AIMS greater than 50 is clearly characterized by independent walking. A complementary analysis is shown in Figure S5, plotting these posture + movement combinations against BIMS levels. It shows highly comparable trajectory shapes, as expected from the high correlation between BIMS and AIMS (Figure 2a) and the extended developmental range that BIMS models.

### AIMS prediction with the MAIJU measurement data

**Figure 4:**
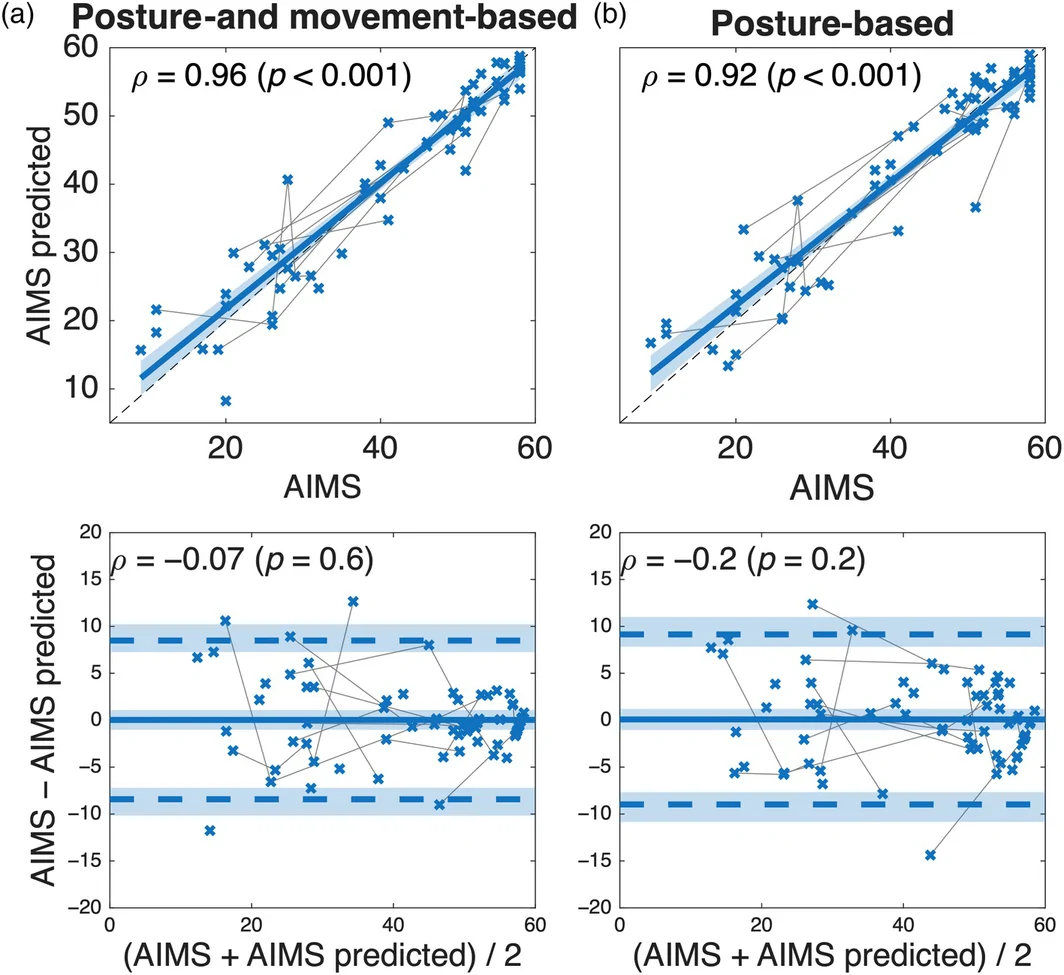
Prediction of AIMS score using the fully automated outputs of the MAIJU analysis pipeline. (a) AIMS prediction based on all posture and movement detection. (b) MAIJU prediction when using posture alone. Both AIMS predictions are very strongly correlated to the expert‐observed AIMS scores; however, use of both posture and movement is significantly better than posture alone. The blue line depicts the best linear regression fit between the original and predicted AIMS scores (the shading shows the 95% confidence intervals and the lines connect repeated assessments in the individual). The Blandt–Altman analyses show the respective comparisons between AIMS assessments using the clinical (AIMS) versus MAIJU‐based (AIMS predicted) results. There was no meaningful bias observed in either version of the AIMS predictions. Abbreviations: AIMS, Alberta Infant Motor Scale; MAIJU, Motor Assessment of Infants with a JUmpsuit.

These results suggest a close link between AIMS and the data measured with the MAIJU wearable suit. Next, we studied to what extent the fully automated analysis pipeline of MAIJU measurements could be used for algorithmic prediction of AIMS scores. By combining all automatically detected posture + movement combinations, we obtained algorithmically predicted AIMS values that showed a strong correlation with the original AIMS scores (rho = 0.96, *p* < 10^−30^; *n* = 67) (Figure 4a). However, AIMS prediction using MAIJU‐derived data was not significantly better than the correlation between BIMS and AIMS (cocor test). In addition, we trained AIMS prediction from the MAIJU‐derived posture detection alone because posture detection can be done more robustly over extended periods of time. The AIMS scores predicted from only posture detection in the MAIJU data still showed a strong correlation with the original AIMS (rho = 0.92, *p* < 10^−20^; *n* = 67) (Figure 4b). However, the posture‐based AIMS prediction had a significantly lower correlation with the AIMS score when compared with the AIMS prediction that used all posture + movement information from the measurement session (all cocor tests: *p* < 0.001). Taken together, our results suggest that MAIJU‐derived posture detection alone can give robust representation of an infant's performance, while detecting movements will add to its accuracy.

### Centile assessment with AIMS versus MAIJU

**Figure 5:**
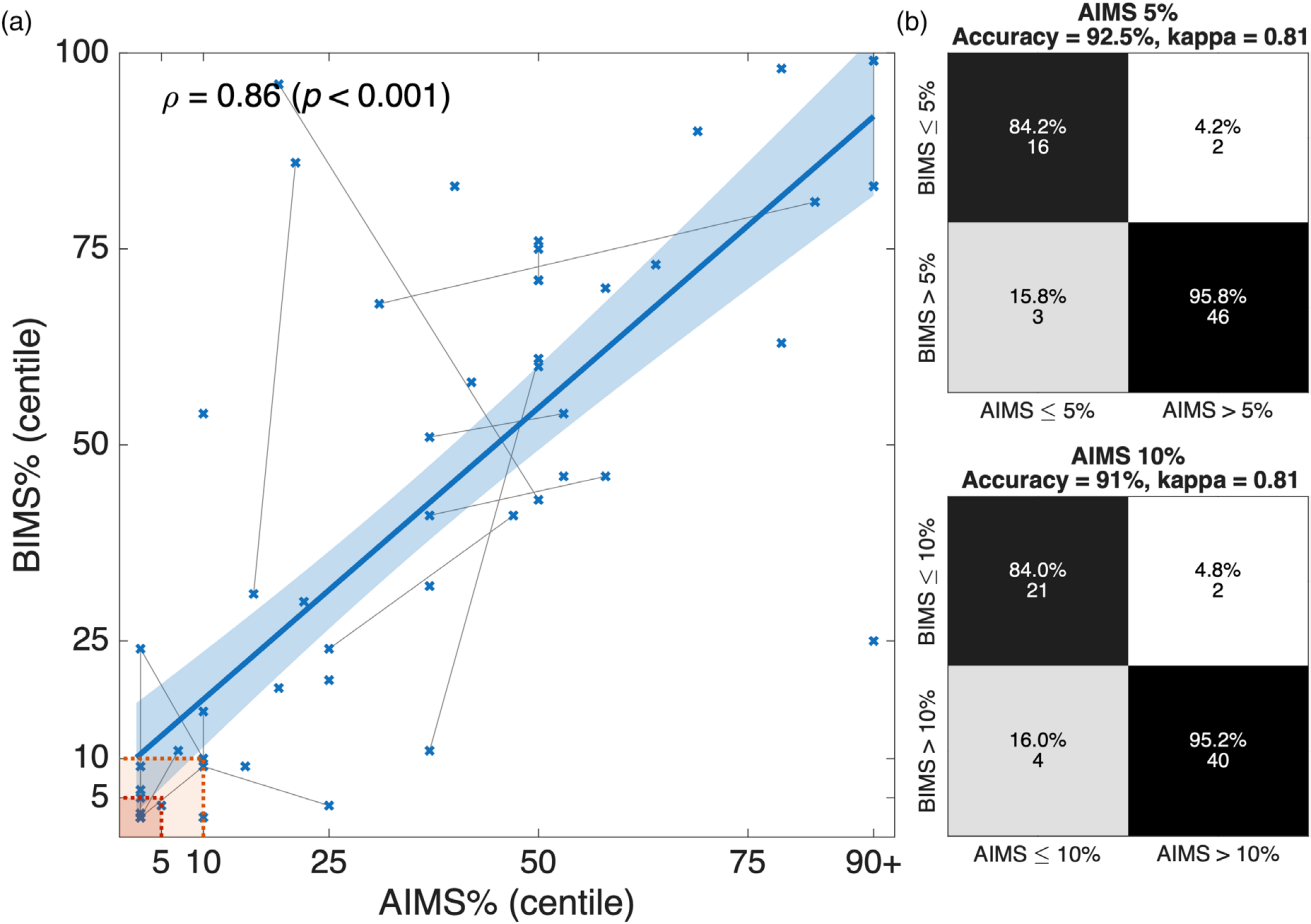
Identifying low‐performing infants with BIMS% versus AIMS%. (a) Scatter plot of BIMS% and AIMS% over the whole cohort. Lower left corner, Strong overall correlation and a clear concentration of low centile cases (dark red <5%; light red < 10%). (b) Confusion matrices depicting accuracy in identifying low‐AIMS% infants using the BIMS%. Abbreviations: AIMS, Alberta Infant Motor Scale; BIMS, BABA Infant Motor Score.

In everyday clinical work, identifying infants for closer follow‐up and therapeutic interventions is commonly done using age‐dependent centile cut‐offs (AIMS%), preferably over multiple assessment time points. In this study, we assessed the potential of the MAIJU‐derived BIMS to identify infants falling below the most often used AIMS% cut‐off levels, that is, less than 5% (*n* = 19) and less than 10% (*n* = 25). Figure 5 shows a scatter plot of all infants for AIMS% versus BIMS%; it shows a strong overall correlation between BIMS% and AIMS% (rho = 0.86, *p* < 10^−20^, *n* = 67; Figure 5a) despite the wider scatter at higher centile values that are relatively uninformative in this context. Most importantly, infants with low AIMS% can be identified with more than 90% (kappa = 0.81) accuracy using either cut‐off (see the confusion matrix in Figure 5b). Performance scores are also nearly identical when controlling for repeated measurements with the same infant by computing the weighted kappa and accuracy scores (AIMS 5% weighted kappa = 0.80, weighted accuracy = 93.6%; AIMS 10% weighted kappa = 0.81, weighted accuracy = 92.5%; see Figure S6 for a complementary BIMS cut‐off of less than 25%).

## DISCUSSION

This study shows that at‐home multi‐sensor wearable measurements with functionally specific analytics provides metrics of motor development that correlate strongly with an independent AIMS assessment. Remarkably, the holistic motor performance measure BIMS emerges naturally from the wearable measured data, without any deliberate attempt to model AIMS or any other clinical assessment of gross motor development. The present findings are fully compatible with prior studies where at‐home wearables were used to track infants' motor development.^8^, ^11^, ^12^, ^24^, ^25^, ^26^ We have extended prior studies by showing concurrent validity and detailed comparison against a widely used clinical assessment method, supporting the idea that a wearable method of this kind could be used as an objective alternative, or complement, to an expert‐performed assessment.

The most striking finding was a strong overall correlation between the MAIJU‐derived holistic score BIMS and the visually observed total AIMS score. While both aim to quantify gross motor development, the functional range in AIMS caps at a lower motor performance level compared to the coverage of BIMS, ranging from lying ‘still supine’ to ‘fluent walking’. Consequently, BIMS levels may still increase in infants after their AIMS has reached its maximum, suggesting that BIMS can support longitudinal follow‐up over a wider functional range. Comparison of AIMS total scores with specific MAIJU‐derived metrics at each measurement session showed clear developmental trajectories, indicating that the automatically computed MAIJU metrics capture the essentials of visual AIMS observations.

While the present results are encouraging, it is important to consider their relationship to the wider wearable literature and practical implications. The present results are built on an accurate and validated detection of infants' posture and movement,^11^ which is only possible from multi‐sensor wearable measurements using fixed sensor locations.^27^ Hence, the present results do not apply to the commonly used single or two‐sensor ‘actigraphy’ set‐ups, where the analytical detail is only focused on the overall activity levels.^28^ Logistically, a multi‐sensor solution like MAIJU is quick and easy to learn compared to the training needed with conventional actigraphy solutions. This is achieved by standardizing measurements with the suit design and its associated intuitive app, while the analysis pipeline operates in fully automated fashion. Recent studies showed a wide scalability in extreme environments, such as the Helsinki metropolitan area and rural Malawi.^29^

The present work has some limitations. The cohort is small; larger cohorts with more longitudinal assessments and in different environments are needed.^30^ Also, the AIMS assessments were collected using a mixed approach of live and video assessments, and the infant cohort was heterogeneous. The present findings indicate a very robust link because both of these caveats could have deflated the correlations between AIMS and MAIJU. Nevertheless, it is important to carry out more systematic prospective data collection in different use cases, different kinds of infant populations, and different geographical environments.^31^

Compared to clinical assessments by trained experts, an assessment with at‐home wearables of this kind may provide clear complementary information. Conceptually, the key difference between AIMS and MAIJU is the way AIMS assesses infants by observing their maximal performance (often guided by the physiotherapist), whereas the MAIJU measures infants' spontaneous activity as it occurs at home. The BIMS and AIMS were convincingly strongly aligned; thus, both the MAIJU metrics and AIMS capture essential trajectories in the motor development of the individual. Practically, the motor metrics from the MAIJU analytics are quantitative and objective, and can be repeated frequently. Repeated assessments are important in the clinical context to mitigate the effects of situational variability; they are also particularly useful in performing fully decentralized intervention studies or other remote neurodevelopmental surveillance.^31^, ^32^ Moreover, a wearable‐based method harmonizes assessments and supports the direct data collation that is often needed in multicentre studies.^31^, ^32^

## Supporting information


**Figure S1:** Histograms for the data set age and AIMS distribution.

**Figure S2:** Relationship between measurement interval and the difference between BIMS versus AIMS.

**Figure S3:** Trajectory modelling and smoothing example.

**Figure S4:** Breakdown of AIMS versus BIMS correlations according to the method of performing AIMS in‐person or by using parentally recorded videos.

**Figure S5:** Developmental trajectories of posture + movement combinations as a function of BIMS.

**Figure S6:** Identifying low‐performing infants with BIMS% <25% versus AIMS% <10%.

## References

[1] World Health Organization . Motor development milestones. 2006 [Available from: https://www.who.int/tools/child‐growth‐standards/standards/motor‐development‐milestones.]

[2] Novak I , Morgan C , Adde L , Blackman J , Boyd RN , Brunstrom‐Hernandez J , et al. Early, Accurate Diagnosis and Early Intervention in Cerebral Palsy: Advances in Diagnosis and Treatment. JAMA Pediatr. 2017;171(9):897–907. 28715518 10.1001/jamapediatrics.2017.1689 PMC9641643

[3] Franchak JM , Kadooka K , Fausey CM . Longitudinal relations between independent walking, body position, and object experiences in home life. Dev Psychol. 2024;60(2):228–42. 38190212 10.1037/dev0001678

[4] Boonzaaijer M , Suir I , Mollema J , Nuysink J , Volman M , Jongmans M . Factors associated with gross motor development from birth to independent walking: A systematic review of longitudinal research. Child Care Health Dev. 2021;47(4):525–61. 33210319 10.1111/cch.12830 PMC8252538

[5] Berger S , Horger M , DeMasi A , Karasik L . Motor Development in Context. Oxford Research Encyclopedia of Psychology. 2021.

[6] Group WHOMGRS . WHO Motor Development Study: windows of achievement for six gross motor development milestones. Acta Paediatr Suppl. 2006;450:86–95. 16817682 10.1111/j.1651-2227.2006.tb02379.x

[7] Darrah J , Senthilselvan A , Magill‐Evans J . Trajectories of serial motor scores of typically developing children: Implications for clinical decision making. Infant Behav Dev. 2009;32(1):72–8. 19081141 10.1016/j.infbeh.2008.10.001

[8] Airaksinen M , Gallen A , Taylor E , de Sena S , Palsa T , Haataja L , et al. Assessing Infant Gross Motor Performance With an At‐Home Wearable. Pediatrics. 2025; 155(4):e2024068647. 40049221 10.1542/peds.2024-068647

[9] Suir I , Boonzaaijer M , Oudgenoeg‐Paz O , van Schie PEM , Nuysink J , Jongmans MJ . Parental Beliefs About the Motor Development of Dutch Infants Born Very Preterm: A Cohort Study. Pediatr Phys Ther. 2024;36(1):95–103. 38227754 10.1097/PEP.0000000000001069

[10] Greenspan B , Cunha AB , Lobo MA . Design and validation of a smart garment to measure positioning practices of parents with young infants. Infant Behav Dev. 2021;62:101530. 33548894 10.1016/j.infbeh.2021.101530 PMC7927978

[11] Airaksinen M , Gallen A , Kivi A , Vijayakrishnan P , Hayrinen T , Ilen E , et al. Intelligent wearable allows out‐of‐the‐lab tracking of developing motor abilities in infants. Commun Med (Lond). 2022;2:69. 35721830 10.1038/s43856-022-00131-6 PMC9200857

[12] Airaksinen M , Taylor E , Gallen A , Ilen E , Saari A , Sankilampi U , et al. Charting infants' motor development at home using a wearable system: validation and comparison to physical growth charts. EBioMedicine. 2023;92:104591. 37137181 10.1016/j.ebiom.2023.104591 PMC10176156

[13] Franchak JM , Tang M , Rousey H , Luo C . Long‐form recording of infant body position in the home using wearable inertial sensors. Behav Res Methods. 2024;56(5):4982–5001. 37723373 10.3758/s13428-023-02236-9

[14] Piper M , Darrah J , Maguire T , Redfern L . Motor Assessment of the developing infant. Saunders; 1994.

[15] Spittle AJ , Doyle LW , Boyd RN . A systematic review of the clinimetric properties of neuromotor assessments for preterm infants during the first year of life. Dev Med Child Neurol. 2008;50(4):254–66. 18190538 10.1111/j.1469-8749.2008.02025.x

[16] Boonzaaijer M , van Dam E , van Haastert IC , Nuysink J . Concurrent Validity Between Live and Home Video Observations Using the Alberta Infant Motor Scale. Pediatr Phys Ther. 2017;29(2):146–51. 28350771 10.1097/PEP.0000000000000363 PMC5374751

[17] Piper MC , Pinnell LE , Darrah J , Maguire T , Byrne PJ . Construction and validation of the Alberta Infant Motor Scale (AIMS). Can J Public Health. 1992;83 Suppl 2:S46–50. 1468050

[18] Mendonca B , Kong M , Coombs A , Kysh L , Sargent B . Psychometric properties of the Alberta Infant Motor Scale and culturally adapted or translated versions when used for infant populations internationally: A systematic review. Dev Med Child Neurol. 2025;67(2):165–76. 39234875 10.1111/dmcn.16070 PMC11695772

[19] Karasik LB , Robinson SR . Milestones or millstones: How standard assessments mask cultural variation and misinform policies aimed at early childhood development. Policy Insights from the Behavioral and Brain Sciences. 2022;9(1):57–94.

[20] Taylor E , Airaksinen M , Gallen A , Immonen T , Ilen E , Palsa T , et al. Quantified Assessment of Infant's Gross Motor Abilities Using a Multisensor Wearable. J Vis Exp. 2024(207). 10.3791/65949 38829118

[21] Gallen A , Taylor E , Salmi J , Haataja L , Vanhatalo S , Airaksinen M . Early gross motor performance is associated with concurrent prelinguistic and social development. Pediatr Res. 2025; 98(3):1016–1022. 39824938 10.1038/s41390-025-03832-5 PMC12507688

[22] de Sena S , Haggman M , Ranta J , Roienko O , Ilen E , Acosta N , et al. NAPping PAnts (NAPPA): An open wearable solution for monitoring Infant's sleeping rhythms, respiration and posture. Heliyon. 2024;10(13):e33295. 39027497 10.1016/j.heliyon.2024.e33295 PMC11255670

[23] Darrah J , Bartlett D , Maguire TO , Avison WR , Lacaze‐Masmonteil T . Have infant gross motor abilities changed in 20 years? A re‐evaluation of the Alberta Infant Motor Scale normative values. Dev Med Child Neurol. 2014;56(9):877–81. 24684556 10.1111/dmcn.12452 PMC4293464

[24] Trujillo‐Priego IA , Lane CJ , Vanderbilt DL , Deng W , Loeb GE , Shida J , et al. Development of a Wearable Sensor Algorithm to Detect the Quantity and Kinematic Characteristics of Infant Arm Movement Bouts Produced across a Full Day in the Natural Environment. Technologies (Basel). 2017;5(3). 10.3390/technologies5030039 PMC5558826 28824853

[25] Deng W , Trujillo‐Priego IA , Smith BA . How Many Days Are Necessary to Represent an Infant's Typical Daily Leg Movement Behavior Using Wearable Sensors? Phys Ther. 2019;99(6):730–8. 31155662 10.1093/ptj/pzz036 PMC6545277

[26] Pini N , Fifer WP , Oh J , Nebeker C , Croff JM , Smith BA , et al. Remote data collection of infant activity and sleep patterns via wearable sensors in the HEALthy Brain and Child Development Study (HBCD). Dev Cogn Neurosci. 2024;69:101446. 39298921 10.1016/j.dcn.2024.101446 PMC11426054

[27] Airaksinen M , Räsänen O , Vanhatalo S . Trade‐offs between simplifying inertial measurement unit‐based movement recordings and the attainability of different levels of analyses: systematic assessment of method variations. JMIR Mhealth Uhealth. 2025 Jun 3;13:e58078. 10.2196/58078 PMC12151449 40460316

[28] Bruijns BA , Truelove S , Johnson AM , Gilliland J , Tucker P . Infants' and toddlers' physical activity and sedentary time as measured by accelerometry: a systematic review and meta‐analysis. Int J Behav Nutr Phys Act. 2020;17(1):14. 32028975 10.1186/s12966-020-0912-4 PMC7006115

[29] Taylor E , Airaksinen M , Ihamuotila R , Kivela M , Ashorn U , Haataja LM , et al. Assessing motor development with wearables in low‐resource settings: feasibility in rural Malawi. Pediatr Res. 2026;99:2444–2450. 40033080 10.1038/s41390-025-03818-3 PMC13485677

[30] Boonzaaijer M , Oudgenoeg‐Paz O , Suir I , Westers P , Nuysink J , Volman M , et al. Modeling a gross motor curve of typically developing Dutch infants from 3.5 to 15.5 months based on the Alberta Infant Motor Scale. Early Hum Dev. 2021;157:105366. 33865116 10.1016/j.earlhumdev.2021.105366

[31] González Barral C , Servais L . Wearable sensors in paediatric neurology. Dev Med Child Neurol. 2025;67:834–853. 39888848 10.1111/dmcn.16239 PMC12134426

[32] Rawnsley KL , Cheong JLY , Bennett K , Mahady ML , Smith SE , Zannino D , et al. Synchronous telehealth and face‐to‐face administration of the Alberta Infant Motor Scale. Dev Med Child Neurol. 2026 Jan;68(1):91–98. 40576266 10.1111/dmcn.16391 PMC12683301

