# Supplementary material for: Gross motor performance of infants with an at‐home wearable measurement and the Alberta Infant Motor Scale: A concurrent validity study
Source: Dev Med Child Neurol. 2026 Feb 16;68(10):1440–9. doi: 10.1111/dmcn.70175 (PMC13545482; doi:10.1111/dmcn.70175)
Supplement: Supplementary file 1 [file DMCN-68-1440-s001.docx]

**Supplemental Figure S5.** Developmental trajectories of P/M combinations as a function of BIMS (X axis). This figure is complementary to the Figure 3, which showed the same P/M development as a function of AIMS scores.
