# Supplementary material for: Gross motor performance of infants with an at‐home wearable measurement and the Alberta Infant Motor Scale: A concurrent validity study
Source: Dev Med Child Neurol. 2026 Feb 16;68(10):1440–9. doi: 10.1111/dmcn.70175 (PMC13545482; doi:10.1111/dmcn.70175)
Supplement: Supplementary file 2 [file DMCN-68-1440-s002.docx]

**Supplementary Figure S3.** Trajectory modeling and smoothing example. Top panels: We utilize the beta distribution (parameters alpha and beta) to model the BIMS and AIMS-based bin statistics (raw; blue; 12 total bins). We perform 3-tap moving average filtering on the alpha and beta parameters (current, 1 previous, and 1 upcoming sample; red). Bottom: Percentiles computed from the raw and smoothed beta distribution parameters.
