# Supplementary material for: Gross motor performance of infants with an at‐home wearable measurement and the Alberta Infant Motor Scale: A concurrent validity study
Source: Dev Med Child Neurol. 2026 Feb 16;68(10):1440–9. doi: 10.1111/dmcn.70175 (PMC13545482; doi:10.1111/dmcn.70175)
Supplement: Supplementary file 3 [file DMCN-68-1440-s003.docx]

**Supplemental Figure S6.** Identifying low performing infants with BIMS-% <25% vs AIMS-% <10%. The confusion matrix shown here is complementary to the ones shown in Figure 5 with lower cutoff levels. As compared to the confusion matrix with cutoff <10% for both BIMS and AIMS (Fig 5B, lower matrix), here we see marginally lower accuracy (88.1 vs 91%) and kappa (0.76 vs 0.81), whereas it identifies a few more infants detected by both BIMS and AIMS.
