# Supplementary material for: Gross motor performance of infants with an at‐home wearable measurement and the Alberta Infant Motor Scale: A concurrent validity study
Source: Dev Med Child Neurol. 2026 Feb 16;68(10):1440–9. doi: 10.1111/dmcn.70175 (PMC13545482; doi:10.1111/dmcn.70175)
Supplement: Supplementary file 4 [file DMCN-68-1440-s004.docx]

**Supplemental Figure S2**. Relationship between measurement interval (BIMS vs AIMS), and the difference between BIMS vs AIMS scores (left) or BIMS-% vs AIMS-% (right). The absence of correlation indicates that the time difference between BIMS and AIMS assessments (X axis) did not affect their mutual relationship, shown to be very strong (Figure 2).
