# Supplementary material for: Gross motor performance of infants with an at‐home wearable measurement and the Alberta Infant Motor Scale: A concurrent validity study
Source: Dev Med Child Neurol. 2026 Feb 16;68(10):1440–9. doi: 10.1111/dmcn.70175 (PMC13545482; doi:10.1111/dmcn.70175)
Supplement: Supplementary file 5 [file DMCN-68-1440-s005.docx]

**Supplemental Figure S4.** Breakdown of AIMS vs BIMS correlations according to the method of performing AIMS in-person (left) or by using parentally recorded videos (right).
